# 74 Hereditary angioedema revealed by compartment syndrome

**DOI:** 10.1093/rheumatology/keac496.070

**Published:** 2022-10-07

**Authors:** N Boutrid, H Rahmoune, H Boutrid, B Bioud, A.S Chehad, M Amrane

**Affiliations:** 1 LMCVGN Laboratory, Faculty of Medicine, Sétif-1 University, Algeria; 2 Pediatrics, Setif University Hospital, Algeria; 3 Faculty of Medicine, Algiers-1 University, Algeria; 4 Faculty of Medicine, Constantine-3 University, Algeria

## Abstract

**Background:**

Hereditary angioedema (HAE) can present with a wide spectrum of clinical symptoms, including articular features like the compartment syndrome.

**Objective:**

We present the peculiar case of a young female patient suffering from this rare complement system disorder and presenting as a « wrist oedema ».

**Methods:**

A 14-year-old girl was admitted in the emergency room during a night shift. She presented with acute compartment (wrist) syndrome due to edematous compression of the median nerve, associated with a typical personal history of HAE. It resolved within 6 h with IV steroids and hyperhydration.

The quantitative and qualitative C1 INH esterase returning negative, the diagnosis of HAE type III was made.

The patient was treated with long-term treatment based on Danazol, (the most frequently prescribed attenuated androgen to treat HAE).

**Results:**

HAE is due to the deficiency or dysfunction of the C1 inhibitor protein (quantitative or qualitative deficiency). A new nomenclature has replaced the initial use of denominations HAE type 1, 2 or 3; to speak rather of HAE with a deficient C1 inhibitor (type 1), with a dysfunctional C1 inhibitor (type 2) or with a normal C1 inhibitor (type 3)

Type 3 HAE is rare and difficult to treat. It may respond to anti-estrogenic drugs such as progestins and especially to androgens; specific (very expensive) molecules do exist and act directly on the bradykinin pathways

**Conclusion:**

A compartment syndrome may reveal extremely rare conditions such as hereditary angioedema.

Early recognition and management are the guarantee of a preserved functional prognosis.

